# Ebselen inhibits QSOX1 enzymatic activity and suppresses invasion of pancreatic and renal cancer cell lines

**DOI:** 10.18632/oncotarget.4099

**Published:** 2015-06-01

**Authors:** Paul D. Hanavan, Chad R. Borges, Benjamin A. Katchman, Douglas O. Faigel, Thai H. Ho, Chen-Ting Ma, Eduard A. Sergienko, Nathalie Meurice, Joachim L. Petit, Douglas F. Lake

**Affiliations:** ^1^ School of Life Sciences, Mayo Clinic Collaborative Research Building, Arizona State University, Scottsdale, AZ, USA; ^2^ Center for Personalized Diagnostics, Biodesign Institute, Arizona State University, Tempe, AZ, USA; ^3^ Mayo Clinic Arizona, Scottsdale, AZ, USA; ^4^ Conrad Prebys Center for Chemical Genomics, Sanford-Burnham Medical Research Institute, La Jolla, CA, USA

**Keywords:** QSOX1, LOPAC^1280^, ebselen, renal cancer, pancreatic cancer

## Abstract

Quiescin sulfhydryl oxidase 1 (QSOX1) is a highly conserved disulfide bond-generating enzyme that is overexpressed in diverse tumor types. Its enzymatic activity promotes the growth and invasion of tumor cells and alters extracellular matrix composition. In a nude mouse-human tumor xenograft model, tumors containing shRNA for QSOX1 grew significantly more slowly than controls, suggesting that QSOX1 supports a proliferative phenotype *in vivo*. High throughput screening experiments identified ebselen as an *in vitro* inhibitor of QSOX1 enzymatic activity. Ebselen treatment of pancreatic and renal cancer cell lines stalled tumor growth and inhibited invasion through Matrigel *in vitro*. Daily oral treatment with ebselen resulted in a 58% reduction in tumor growth in mice bearing human pancreatic tumor xenografts compared to controls. Mass spectrometric analysis of ebselen-treated QSOX1 mechanistically revealed that C165 and C237 of QSOX1 covalently bound to ebselen. This report details the anti-neoplastic properties of ebselen in pancreatic and renal cancer cell lines. The results here offer a “proof-of-principle” that enzymatic inhibition of QSOX1 may have clinical relevancy.

## INTRODUCTION

QSOX1 is a highly conserved FAD-dependent sulfhydryl oxidase that catalyzes disulfide bond formation in reduced proteins and sulfhydryl-containing small molecules. QSOX1 has been reported to be overexpressed in diverse tumor types such as pancreatic [1, 2], breast [3, 4], and prostate cancers [5], and is associated with a proliferative and invasive phenotype [2, 3, 6]. QSOX1 expression is not detected in normal breast tissue and is positively correlated with high grade breast tumors by transcriptomic analyses of EST and SAGE databases, as well as quantitative PCR of cDNA isolated from normal and malignant breast tissue [4]. Increased QSOX1 expression in luminal B breast cancers predicts poor overall and relapse-free survival [3]. QSOX1 RNA is elevated when expression of the transcriptional regulator NKX3.1 is reduced in prostatic intraepithelial neoplasia [5], suggesting that QSOX1 expression may contribute to prostate cancer progression.

The predominant isoforms of QSOX1, QSOX-L (long, 742a.a.) and QSOX1-S (short, 604a.a.) have intra- and extracellular functions related to growth, invasion, and extracellular matrix structure and function. In pancreatic and breast cancer cell lines, QSOX1 expression is associated with a highly invasive phenotype and the increased activation of matrix metalloproteinase -2 and -9 [2, 3]. QSOX1 is required for the proper incorporation of laminin α4 subunits into the extracellular matrix (ECM), and reducing QSOX1 expression decreases cell adhesion and increases sulfhydryls in the ECM [7].

Here we report that QSOX1 expression contributes to tumor growth in an *in vivo* pancreatic tumor cell xenograft model, supporting QSOX1 as a potential molecular therapeutic target. A high-throughput screen of the small molecule LOPAC^1280^ library identified ebselen as a inhibitor of QSOX1 enzymatic activity *in vitro*. In this study, we investigate the biological effects of QSOX1 inhibition and demonstrate, mechanistically, how ebselen suppresses QSOX1 activity.

## RESULTS

### QSOX1 expression drives increased tumor growth *in vivo*

**Figure 1:**
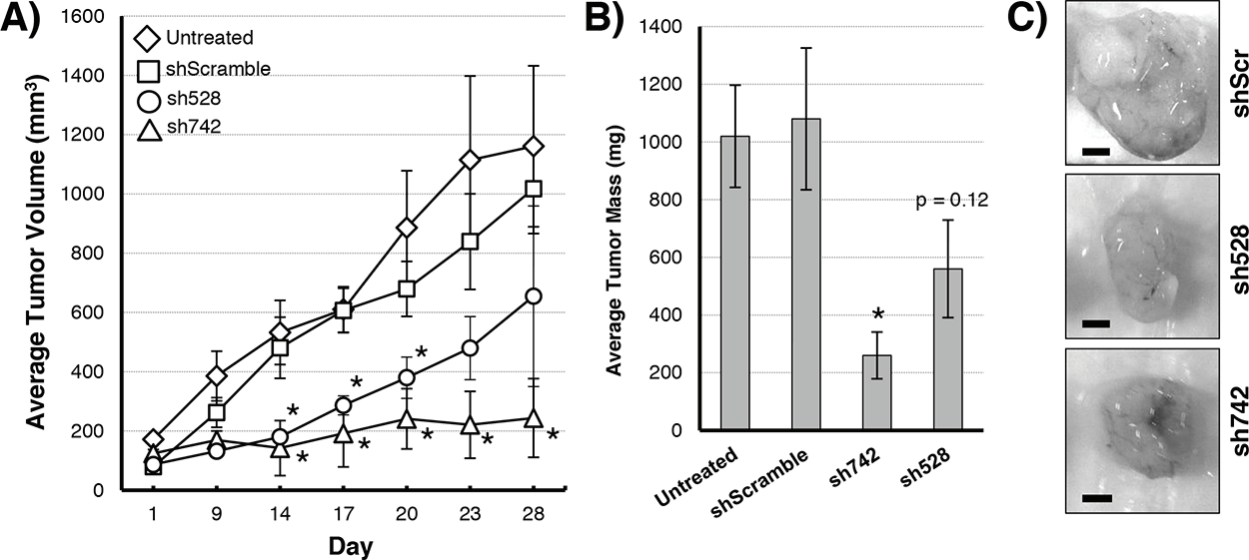
Growth of MIAPaCa-2 tumors over 28 days in nude mice **A.** Growth kinetics of MiaPaca-2 tumors with QSOX1 knockdowns (sh742 triangles, sh528 circles), scrambled control (squares), or untreated (diamonds). **B.** Final tumor masses on day 28. **C.** Images of shScr (top), sh528 (middle), or sh742 (bottom) tumors dissected from mice on day 28. Significance was determined by *T*-test; knockdowns were compared to vehicle-treated tumors. *p < 0.05. Bar = 3 mm.

We hypothesized that suppressing QSOX1 levels in tumors expressing shRNAs specific for QSOX1 would slow their growth compared untreated and scrambled shRNA control (shScr) cells. Tumors containing QSOX1 shRNAs (sh742 or sh528) grew at a reduced rate compared to shScr control xenografts (Figure 1A). Tumor masses on day 28 of the experiment showed that tumor growth was reduced by 77% in tumors transduced with sh742, and by 48% in tumors transduced with sh528 compared to shScr tumors (Figure 1B, 1C). These results indicate that QSOX1 expression promotes tumor growth *in vivo* and suggest that QSOX1 could be a target for potential anti-neoplastic compounds.

### Ebselen inhibits rQSOX1 activity *in vitro*

We used a sulfhydryl activity assay similar to the one developed by Colin Thorpe's group [8] to screen recombinant QSOX1 against a library of pharmacologically active compounds, LOPAC^1280^. In this enzymatic assay (Supplementary Figure S1A), rQSOX1 oxidizes a reduced RNAse A or DTT substrate, producing H_2_O_2_ detected by a luminescent reaction. In the presence of a QSOX1 inhibitor, the sulfhydryl oxidase activity of QSOX1 is blocked, preventing disulfide bond formation and H_2_O_2_ production. The relative inhibitory activity of LOPAC^1280^ is plotted in Figure S1B. We identified ebselen (Figure S1C) as an inhibitor of QSOX1 enzyme activity, with greater inhibitory activity against QSOX1 than GOx (Figure S1D); the IC_50_ for ebselen inhibition of QSOX1 and GOx was determined to be 5.4 μM and 20.5 μM, respectively. Confirmation of ebselen's inhibitory activity was obtained by HyPerBlu luminescent detection (Figure S1D, middle plot) and HVA-based fluorescent assays showing decreased fluorescence as inhibitor concentration increases (Figure S2).

### Ebselen reduces tumor cell invasion

One of the fundamental properties of malignant cells leading to metastatic disease is invasion. Since ebselen inhibits QSOX1, we hypothesized that it would suppress invasion of tumor cells similar to shRNA-mediated knockdown of QSOX1.

**Figure 2:**
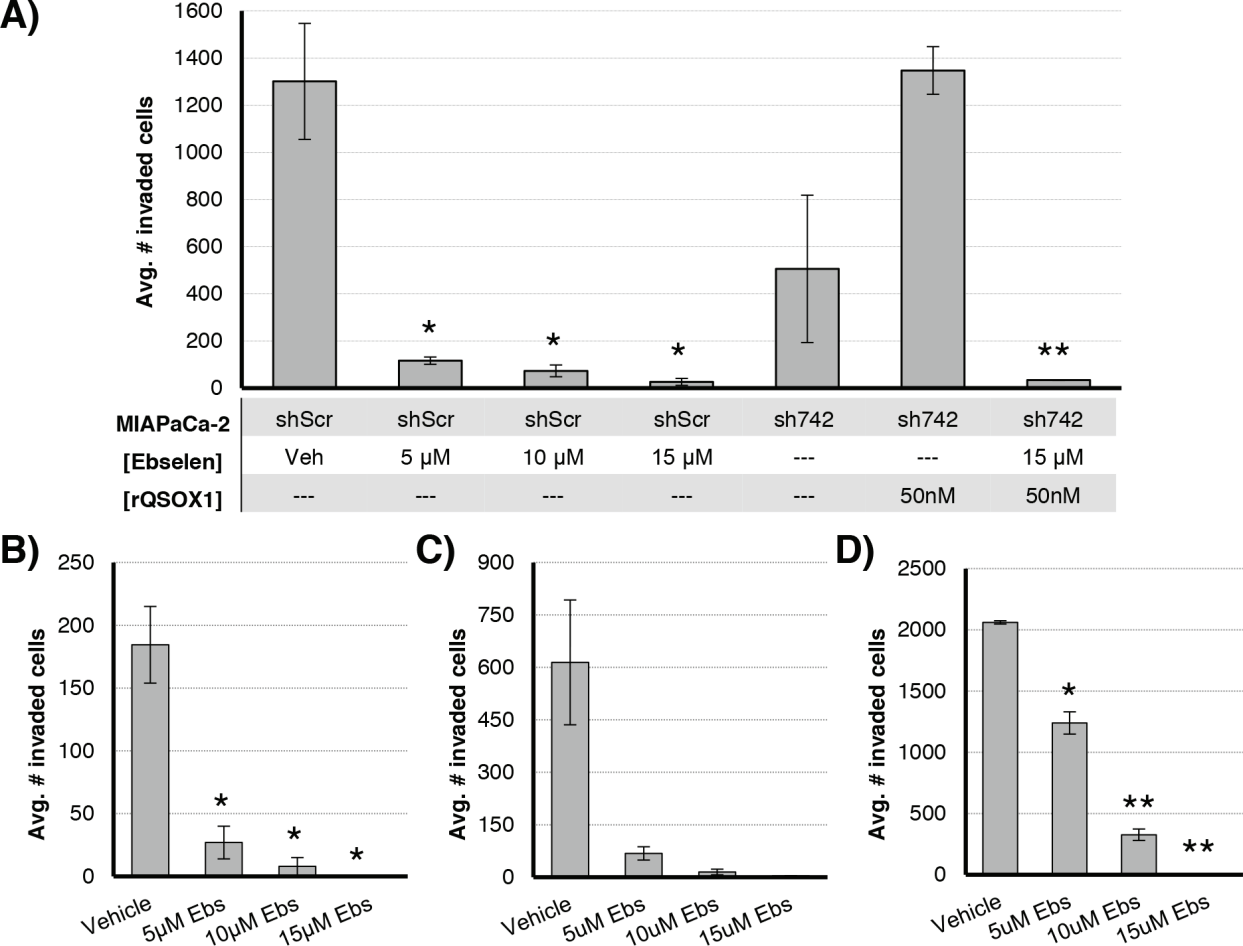
Ebselen inhibits invasion of pancreatic and renal cell cancer cell lines through a Matrigel basement membrane **A.** Isogenic MIAPaCa-2 cell lines transduced with a QSOX1-specific shRNA (sh742) or a nonspecific sequence (shScr) were incubated for 20 hours in invasion well inserts; cells were exposed to 5–15 uM ebselen or vehicle +/− 50 nM rQSOX1 (sh742 only). Invasion of **B.** BXPC3, **C.** 786-O, and **D.** UOK117 cells exposed to ebselen or vehicle. Vehicle was 0.15% DMSO. Error bars represent SEM. For A significance for ebselen-treated shScr cells was calculated for vehicle-treated shScr cells. For sh742 cells, significance is relative to sh742 cells treated with rQSOX1 alone. Statistical significance was determined by *T*-test. *p < 0.05, **p < 0.01.

MIAPaCa-2, BXPC3, 786-O, and UOK117 cells were incubated in matrigel-coated invasion chambers in serum-free media in the presence of ebselen or vehicle. Invading cells were quantified after 20 hours (Figure 2). Isogenic MIAPaCa-2 lines were generated that express shRNAs specific for QSOX1 (sh742 and sh528) or a nonspecific sequence (shScr) (Figure 2A). shScr cells exposed to 5 μM – 15 μM ebselen showed decreased invasion compared to DMSO vehicle-treated cells, with reductions of 91%, 94%, and 98%, respectively. sh742 cells showed greater than 60% decreased invasion compared to shScr cells. Invasion was rescued to levels of vehicle-treated shScr wells when 50 nM active rQSOX1 was added to sh742 wells at the initiation of the assay (Figure 2A, sixth bar). rQSOX1 pre-incubated with 15 μM ebselen and then added to sh742 cells, however, did not rescue invasion. These results suggest that one of the mechanisms by which ebselen suppresses invasion is through QSOX1 inhibition.

We also quantified invasion in ebselen-treated BXPC3, 786-O, and UOK117 cells (Figure 2B–2D). Ebselen suppressed invasion in these tumor cell lines in a concentration-dependent manner. At 5 μM, BXPC3 invasion was reduced by 85%, 89% for 786-O, and 40% for UOK117. 10 μM ebselen treatment decreased BXPC3, 786-O, and UOK117 invasion by 95%, 97%, and 80% compared to vehicle-treated cells, respectively. Near total inhibition of invasion was observed for each cell line treated with 15 μM ebselen. These results were statistically significant in BXPC3 and UOK117 with *p*-values calculated at < 0.05. Results for 786-O were not statistically significant (*p* = 0.08 – 0.09), but show a similar dose-response.

### Ebselen reduces tumor growth *in vivo*

**Figure 3:**
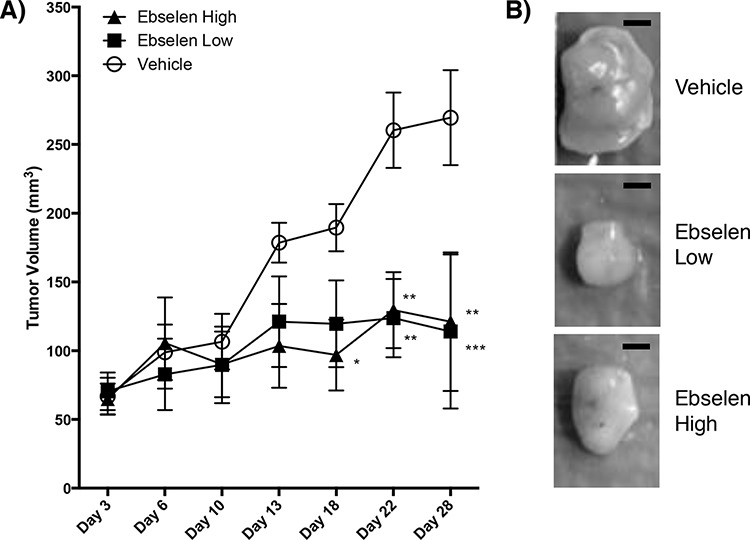
Ebselen treatment of nude mice bearing human tumors **A.** One million MIAPaCa2 cells were mixed with Matrigel and used to inoculate nude mice (5 mice/group) on day 0. Mice were gavaged daily starting on day 3 with vehicle (20% DMSO, open circle), 160 μg/day ebselen (filled square) or 640 μg/day ebselen (filled triangle) in DMSO. Tumor measurements are shown for days 3, 6, 10, 13, 18, 22 and 28. Tumor volume is shown on the Y-axis and time in days is shown on the X-axis. Significance was determined by two-way ANOVA with Dunnet's Test used to correct for multiple comparisons compared to vehicle-treated mice. *p < 0.05, **p < 0.01, ***p < 0.001. **B.** Representative tumors from vehicle-treated and ebselen-treated mice. Bar = 2 mm.

Nude mice subcutaneously injected with MIAPaCa-2 cells were treated for 28 days with ebselen by oral gavage at two clinically achievable doses to determine if ebselen suppresses tumor growth *in vivo*. As shown in Figure 3, daily treatment with ebselen at both high (640 μg) and low (160 μg) doses suppressed tumor growth in MIAPaCa-2 nude mouse xenografts. There was no difference in tumor size between high and low doses, but tumors in mice treated with 160 μg ebselen were ~56% smaller than vehicle-treated mice at day 28. There was no difference in the average mouse weights between the vehicle and ebselen-treated groups (Figure S3), suggesting that the observed difference in tumor growth were not due decreased appetite or decreased nutrient absorption in ebselen-treated mice. Taken together, these results suggest that ebselen decreases tumor growth *in vivo*.

### Ebselen covalently binds to QSOX1

**Figure 4:**
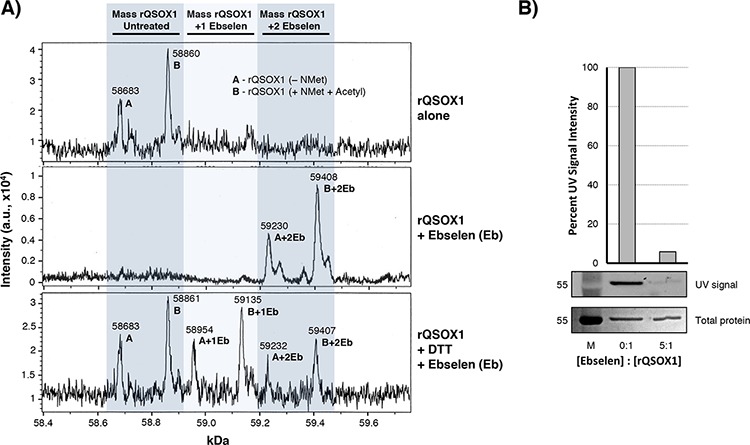
Ebselen binds covalently to rQSOX1 at cysteine residues **A.** Charge deconvoluted ESI-LC-MS spectra of rQSOX (top spectrum) in the absence of substrate, rQSOX1 treated with 5 μM ebselen (middle spectrum), and rQSOX1 treated with 5 μM ebselen in the presence of DTT substrate (bottom spectrum). The mass of an ebselen adduct is 274.18 Da. The left shaded column indicates the mass range of unmodified rQSOX1. Peak A is the mass of rQSOX1 without the N-terminal methionine and peak B is the mass of rQSOX1 with N-acetyl Met. The middle shaded column represents the mass of rQSOX1 with a single bound ebselen molecule with peaks labeled A+1Eb and B+1Eb. The right shaded column represents the mass of rQSOX1 with two ebselen adducts (A+2Eb and B+2Eb). **B.** QSOX1 pretreated with ebselen blocks the binding of fluoresceinated maleimide. A 5-fold molar excess of ebselen was added to 5 μg rQSOX1 prior to maleimide addition. UV imaging of SDS-PAGE gels show that maleimide binding to rQSOX1 is blocked by the addition of ebselen.

Ebselen is reactive with reduced cysteines through the formation of a Se-S bond with target sulfhydryls [9]. We therefore hypothesized that ebselen covalently binds with cysteines in QSOX1, which would suggest a mechanism for the inhibition of enzymatic activity. The formation of ebselen adducts would be expected to increase the mass of QSOX1 by the molecular weight of one or more ebselen molecules, 274.18 Da. We performed LC-MS analysis on untreated or ebselen-treated rQSOX1 in the presence or absence of DTT (an established substrate for QSOX1) [8, 10, 11] (Figure 4A). Untreated rQSOX1 (Figure 4A, top spectrum) displays two prominent peaks with masses of 58683 and 58860 Da, designated “A” and “B,” respectively, corresponding to two post-translationally modified forms of rQSOX1.

Treatment of QSOX1 with ebselen shows a mass shift corresponding to two ebselen adducts per peak (Figure 4A, middle spectrum). In the bottom spectrum of Figure 4A, rQSOX1 was also treated with ebselen in the presence of DTT substrate. The masses of unmodified rQSOX and rQSOX1 containing 1 or 2 ebselen molecules were detected simultaneously, suggesting that DTT can remove ebselen from QSOX1.

We performed a protection assay (Figure 4B), blocking free cysteines in QSOX1 with ebselen pre-treatment followed by incubation with the thiol-reactive compound maleimide. Vehicle-treated rQSOX1 showed strong fluorescence at the expected MW of 58.8 kDa, but ebselen pre-treatment decreased resting QSOX1 fluorescence by > 94%. These results suggest that ebselen binds to free cysteines in resting QSOX1.

### Identification of ebselen-binding cysteines in QSOX1

**Figure 5:**
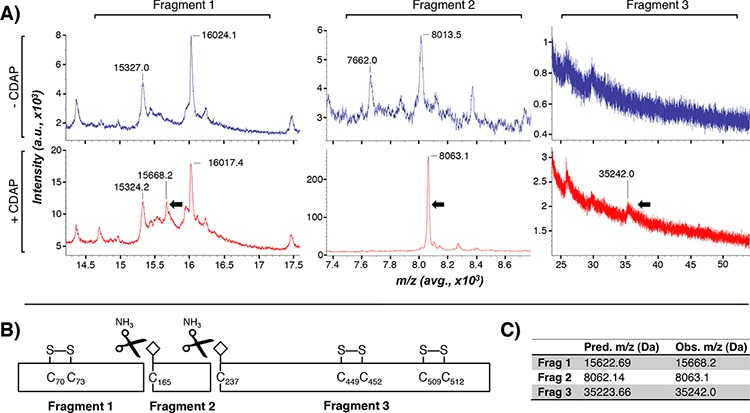
Identification of ebselen-binding cysteines in QSOX1 **A.** Cyanylated rQSOX was cleaved by NH_3_ treatment and reduced with TCEP. Analysis by MALDI-MS identified two cleavage sites that produced QSOX1 fragments with masses of 15 668 Da (Fragment 1), 8 063.1 Da (Fragment 2), and 35 242 Da (Fragment 3). The top and bottom spectra panels in (blue) show untreated (blue) and CDAP-treated (red) QSOX1 peaks, respectively. Arrows indicate unique peaks appearing in CDAP-treated, but not untreated, rQSOX1 samples. **B.** Mapped QSOX1 CDAP cleavage fragments. Peak masses correspond to cysteines 165 and 237 in wild-type QSOX1. Cyanyl groups are depicted as white diamonds. Three redox-active C-X-X-C pairs are shown disulfide bonded. The four remaining disulfide-bonded cysteines are not shown. Cleavage of rQSOX1 by ammonia (black scissors) produced the three CDAP fragments observed in A. **C.** Predicted and observed average m/z for cleavage at residues C165 and C237. The predicted masses for fragments 2 and 3 include an additional 25 Da from the cyanyl group.

We employed a cyanylation strategy to identify ebselen-binding cysteines in resting QSOX1, utilizing the reagent 1-cyano-4-dimethylaminopyridinium tetrafluoroborate (CDAP). CDAP cyanylates free, but not disulfide-bound, cysteines in proteins; they are then cleaved on the amino side of the CDAP-cysteine adduct when subjected to alkaline conditions [12–15]. MALDI analysis of CDAP-treated, cleaved and reduced rQSOX1 revealed 3 unique cleavage fragments (Figure 5A). Fragments 1, 2, and 3 had observed masses of 15668, 8063.1, and 35242 Da, respectively (black arrows in Figure 5A). These masses correspond to cleavage with the expected iminothiazolidine formation [12–15] on the N-terminal side of cysteines 165 and 237 in QSOX1 as shown in the diagram in Figure 5B. Predicted versus observed molecular weights for these cleavage products are shown in Figure 5C. The cyanyl group increases fragment masses by 25 Da (fragments 2 and 3).

## DISCUSSION

QSOX1 is an emerging target in tumor biology given its roles in cell growth, invasion, and the regulation of extracellular matrix composition [2–4, 7, 11, 16]. Secreted QSOX1 acts directly in the extracellular matrix to enhance tumorigenic processes through poorly understood mechanisms. Inhibition of QSOX1 activity in the clinic has the potential to decrease the growth and invasion of tumors, possibly containing primary growths and increasing survival.

Here we establish that QSOX1 promotes tumor growth *in vivo*, strengthening the connection between elevated QSOX1 expression and tumor aggression [3–5]. Xenografted MIAPaCa-2 tumors with stable QSOX1 knockdowns grew at significantly reduced rates compared to controls (Figure 1). These data support other studies demonstrating a pro-tumorigenic role for QSOX1 [2–7, 11, 16].

The enzymatic activity of QSOX1 is essential for its roles in invasion and regulation of extracellular matrix composition [3, 7]. An inhibitory antibody recently developed blocks QSOX1 activity and reduces the invasion of tumor cells across a fibroblast monolayer [7], supporting the idea that directly targeting QSOX1 enzyme activity has anti-invasive properties. Thus, small molecule inhibition of QSOX1 may have therapeutic efficacy or reveal novel pathways in QSOX1 biology.

In a high throughput screen of the LOPAC^1280^ chemical library utilizing recombinant QSOX1, we identified ebselen as an inhibitor of QSOX1 enzyme activity. We used *Aspergillus niger* glucose oxidase (GOx) as a general counter-screen to ensure that inhibition was specific for QSOX1. Although both GOx and QSOX1 contain FAD as a cofactor, the former uses FAD as the initial electron acceptor [17], while FAD serves as terminal electron acceptor in the QSOX1 Erv1 domain. In addition, the sequence and structure of the two proteins are very different [18], sharing only 20% sequence identity. Thus the majority of genuine inhibitors are expected to show strong preference for QSOX1. As seen in supplementary Figure S1D, GOx is inhibited by ebselen only at a concentration 4-fold higher than was observed for QSOX1.

Since ebselen reacts with free cysteines and both QSOX1 substrates and its own redox activity are dependent on sulfhydryls, we were initially concerned that the interaction of ebselen with QSOX1 substrates DTT and RNAse A might make ebselen appear to inhibit QSOX1 spuriously through substrate depletion. We used between 150 and 2400-fold molar excesses of substrate thiols over ebselen in confirmatory activity assays to guard against this possibility (Figure S2). If the interaction of ebselen with substrate was extensive, even with total exhaustion of ebselen sufficient unreacted substrate would be available for QSOX1 oxidation. These conditions would allow for near-maximum signal to be detected, preventing the identification of compounds with an indiscriminate preference for free cysteines. Additionally, rQSOX1 enzyme was always added last to reactions such that ebselen was present with substrate before QSOX1 addition. Therefore, the excess substrate would deplete available ebselen prior to the addition of active enzyme.

Ebselen treatment of tumor cell lines resulted in significantly decreased invasion in trans-well invasion assays compared to DMSO vehicle-treated cells (Figure 2). These results are consistent with decreased invasion in cells expressing QSOX1-specific shRNAs [2, 3, 7]. Importantly, rescue of invasion in QSOX1-knockdown cells was achieved with the addition of 50 nM exogenous recombinant QSOX1 enzyme (Figure 2A, fifth bar). However, pre-incubation of recombinant enzyme with 15 μM ebselen prior to addition of QSOX1 to tumor cells did not restore invasive activity (Figure 2A, sixth bar), suggesting that ebselen inactivates QSOX1. In fact, tumor cell invasion was further decreased compared to the sh742 knockdowns, underscoring the incomplete suppression of gene expression using a shRNA system. These results indicate that one mechanism by which ebselen decreases tumor cell invasion is via QSOX1 enzymatic inhibition.

We investigated the growth modulatory effects of ebselen in pancreatic and kidney cancer cell lines (Figure S4). Ebselen was a poor inhibitor of growth in kidney cancer cell lines, but did significantly inhibit growth of pancreatic cell line MIAPaCa-2 at 10 μM and 15 μM and BXPC3 at 15 μM. QSOX1 expression was similar for the cell lines tested by western blotting (Figure S5), thus QSOX1 expression alone fails to explain the growth effects observed. Viability determinations showed that ebselen is not cytotoxic to tumor cells (Figure S6), so decreased growth is therefore attributable to reduced proliferation. Ebselen treatment also causes tumor cells to “round up” (data not shown). While anecdotal, this observation is consistent with the morphological changes observed when QSOX1 expression is suppressed by shRNAs [2, 3].

Incubation of 15 μM ebselen with normal lymphocytes and non-malignant fibroblasts does not result in toxicity (~98% viability after 48 hours, data not shown). Additionally, ebselen has an established safety profile and dosing in humans in phase II clinical trials for cerebral infarct [19–21]. Patients in this 1998 clinical trial who received ebselen had no statistical increase in adverse events compared to placebo groups for new cerebral infarction, new hemorrhagic infarction, gastrointestinal bleeding, nausea/vomiting, or respiratory infection. The authors also note that ebselen treatment did not contribute to the death of any patient [21].

Daily oral gavage of ebselen in human pancreatic tumor xenografts resulted in slower tumor growth than vehicle controls. Ebselen appears to suppress invasion and is not directly cytotoxic, but none-the-less affects human pancreatic tumor cell growth *in vivo* as shown in Figure 3. While both low (160 μg/day) and high (640 μg/day) doses of ebselen decreased tumor growth in our xenograft model, we did not observe differences in tumor volume between dosages. There may be no additional benefit to treatment with higher doses of ebselen beyond a certain threshold. Ebselen treatment did not cause weight loss, independent of tumor size (Figure S3). While the mechanism of decreased tumor cell growth with ebselen treatment may be unclear, one explanation for the decrease in tumor growth in treated mice is inhibition of QSOX1 activity in the extracellular matrix. QSOX1 is required for incorporation of laminin α4 chains in the ECM [7]. Its inhibition may limit the ability of tumor cells to modify the ECM to promote tumor growth. QSOX1 also activates MMP-2 and -9, supporting the role of QSOX1 in forming a pro-tumorigenic microenvironment.

Ebselen is a heterocyclic selenoorganic compound first identified as a glutathione peroxidase mimic and scavenger of organic hydroperoxides [22–24]. The intriguing enzyme-like activity of ebselen forms the basis for its pharmacological effects [25, 26] that include potent antioxidant and anti-inflammatory properties [9, 27]. Ebselen covalently binds to thiols and this has emerged as a mechanism for its activity [28]. Ebselen reacts via reduction of the Se-N bond in the selenazole ring structure, forming a sulfur-selenium bond with target cysteines [29]. We show that ebselen inhibits QSOX1 activity through covalent modification of non-redox cysteines C165 and C237 in the extant thioredoxin-2 domain.

Crystal structures of human rQSOX1 show that its cysteines exist as disulfide pairs in the resting enzyme except for cysteines 165 and 237 [30, 31]. How ebselen inhibits QSOX1 through interaction with these residues is unclear because they are not thought to participate in the accepted disulfide relay mechanism. There is no evidence supporting cysteines other than the redox-active C-X-X-C motifs in the Trx1 and Erv domains as contributing to QSOX1 activity [11, 18, 32–34]. C237 is likely protonated and relatively inert to redox reactions since there are no nearby basic residues to stabilize a thiolate anion [35]. The location of C165 in a predicted disordered region of QSOX1 between the Trx1 and Trx2 domains, however, may allow for interactions with nearby basic resides [31]. A recently described mechanism proposes that the flexible domain architecture of QSOX1 is critical in allowing Trx1 to come into close contact with Erv to transfer electrons to the C-X-X-C in this domain [30, 36]. Ebselen bound to C165 and C237 may interfere with the conformational change required for the interaction of the Trx1 and Erv domains. Another possibility is that these cysteines modulate the activity of QSOX1; C165 is conserved in QSOX1 among vertebrates but not invertebrates (Figure S7) and may have evolved as a mechanism to regulate QSOX1 function. Further enzymatic and structural studies will address these hypotheses.

We have provided evidence that inhibition of QSOX1 activity by ebselen leads to significantly decreased invasion of tumor cell lines *in vitro* and reduced tumor growth *in vitro* and *in vivo*, effects comparable to QSOX1 knockdown. Since metastasis is the cause of most cancer deaths, even partially suppressing invasive processes through QSOX1 inhibition may help prolong survival. This study further establishes QSOX1 as a tractable target for anti-neoplastic drugs. Future studies will identify more potent and specific inhibitors of QSOX1 that may decrease metastasis *in vivo*.

## MATERIALS AND METHODS

### Cell culture

All cells were cultured in a humidified incubator at 37°C with 5% CO_2_. Media was supplemented with 5% fetal bovine serum (Gibco, Carlsbad, CA USA) and 100 μg/ml penicillin/streptomycin (Gibco) Pancreatic tumor cell lines MIAPaCa-2 and BXPC3 were maintained in Dulbecco's Modified Eagle Medium (DMEM), (Gibco). Renal cancer cell line 786-O was cultured in RPMI 1640 (MediaTech, Manassas, VA USA). Renal cancer cell line UOK117 was cultured in DMEM, supplemented with 1X MEM nonessential amino acids (Mediatech). 786-O cells were cultured from frozen stocks purchased from Sigma-Aldrich and were authenticated by STR analysis after thawing. UOK117 cells were received from the Linehan laboratory at the National Cancer Institute and experiments performed from early passage stocks. MIAPaCa-2 and BXPC3 cells were cultured from frozen stocks that underwent independent STR testing 12/2012.

### QSOX1 knockdown

Lentiviruses packaged with short hairpin RNA (shRNA) constructs specific for human QSOX1 (sh528, sh742) or a nonspecific scrambled sequence (shScr) were produced in 293T cells as reported by Katchman et al. [2]. Knockdown of QSOX1 in MIAPaCa-2 was confirmed by western blotting using anti-QSOX1 antibody (Protein Tech, Chicago, IL USA).

### Nude mouse-human tumor xenograft model

Stably transduced MIAPaCa-2 cells (shScr, sh742, and sh528) were harvested from subconfluent cultures after brief exposure to Cell Stripper (Corning, Corning NY). Cell suspensions were counted, washed once in ice cold serum-free RPMI and resuspended in cold 1x PBS. 2.4 × 10^7^ total cells were mixed in a 1:1 ratio with Matrigel (BD Biosciences) according to manufacturer specifications. Cells were kept on ice throughout the procedure. Female athymic Foxn1^nu^ mice (Harlan, Indianapolis, IN USA) were injected with 1 × 10^6^ MIAPaCa-2 cells in Matrigel. Mice were housed in a barrier facility on HEPA-filtered racks. All experiments were conducted with strict adherence to aseptic technique and IACUC regulations. Each mouse was subcutaneously injected with 200 μl of cell suspension into the right hind flank using a 21-gauge needle. Mice were examined every other day to determine the volume of the tumor using calibrated calipers. Real-time tumor volume was measured as V = 0.5 × length × width^2^. When the tumors reached a volume of 1500–2000 mm^3^ the mice were sacrificed and the tumor was excised, measured and weighed.

For ebselen treatment of nude mice, three groups were tested: 1) 20% DMSO (vehicle), 2) 160 μg/day ebselen, and 3) 640 μg/day ebselen. 160 and 640 μg ebselen represent an equivalent dose of 150mg and 600mg for a 70 kg human, respectively. 1 × 10^6^ MIAPaCa-2 cells were injected subcutaneously into each mouse as before, and tumors were allowed to grow for 3 days. Ebselen was then administered once daily through oral gavage for 28 days. Real-time tumor volume was determined through caliper measurement of tumors over the course of the study [37]. Error is represented as SEM. Statistical significance was determined using two-way analysis of variance (ANOVA), comparing drug-treated with vehicle-treated mice. Corrections for multiple comparisons were made using Dunnett's Test.

### rQSOX1 expression and purification

rQSOX1 was produced and purified according to the method of Heckler [32]. Rosetta-gami B (DE3) cells (Novagen, Billerica MA USA) were transformed with 50 ng pET15b containing truncated QSOX1-S. The rQSOX1 construct contains an N-terminal poly-histidine tag and encodes amino acids 33–546 of QSOX1-S. The activity of the recombinant enzyme verified as described below using a dithiothreitol (DTT) substrate [8]. The elution profile for rQSOX1 and MALDI/LC-MS^2^ analysis of trypsin-digested rQSOX are shown in Supplementary Figures S8 and S9, respectively.

### rQSOX1 small molecule screen

A HTS assay was developed using reduced denatured RNAse A substrate [38]. Hydrogen peroxide produced in the QSOX1 reaction was detected using ROS-Glo kit (Promega, Madison WI) as a primary assay and HyPerBlu (Lumigen, Southfield MI) as a secondary assay per manufacturers instructions. Assays were optimized and reaction kinetic parameters were determined. The assays were miniaturized to final volume of 2 uL. HTS was also performed using the ROS-Glo assay with a substrate concentration of 80 uM RNAse A (that is close to K_m_ value of 122 μM). rQSOX1 protein was utilized at 10 nM concentration, > 20-fold over the limit of assay detection yet still on the linear portion of the enzyme-dependent activity. rQSOX1 was screened against compounds from the LOPAC^1280^ library (Sigma-Aldrich, St. Louis MO USA) at 12.5 μM compound concentration. Compounds that demonstrated > 50% inhibition were re-tested using single-concentration in triplicate wells, followed by concentration-dependent confirmation in the primary and secondary QSOX1 assays. A luminescent assay for glucose oxidase (GOx) was used as a counter-screen. The GOx assay utilized glucose as a substrate was detected using ROS-Glo. Active and selective compounds were purchased in dry powder form, dissolved in DMSO and reconfirmed in the assays before use in the confirmatory assays utilizing the GOx counter-screen.

### rQSOX1 activity assay

The sulfhydryl oxidase activity of rQSOX1 was confirmed using DTT and RNAse A substrates and a fluorogenic hydrogen peroxide indicator, homovanilic acid (HVA) [8]. In this assay, 150 nM rQSOX1 was added to 600 μM thiols from reduced DTT or RNAse A, 1 mM HVA, 1.4 μM HRP, and 300 μM EDTA in PBS at 25°C, pH 7.5. Assays were performed in black 96-well plates with a final reaction volume of 150 μl. Fluorescence signal was measured over 10 minutes at λ_ex_ 320 nm and λ_em_ 420 nm using a FlexStation spectrophotometer (Molecular Devices, Sunnyvale CA USA). Readings were taken in 20 second intervals after the addition of rQSOX1. Ebselen was added to reactions at least 10 minutes prior to the addition of rQSOX1 at concentrations ranging from 250 nM – 4 μM.

Results for the HVA-based activity assay for rQSOX1 and ebselen are shown in Supplementary Figure S2.

### Compound

2-phenyl-1, 2-benzisoselenazol-3(2H)-one, ebselen (Sigma-Aldrich), was dissolved in tissue culture-grade DMSO (Sigma-Aldrich, St. Louis, MO.) to a stock concentration of 10 mM and stored at −20°C protected from light.

### Growth kinetics of ebselen-treated tumor cells

1 × 10^4^ cells/well MIAPaCa-2, BXPC3, 786-O, and UOK1117 were plated in duplicate in 24-well plates. Cells were adhered overnight prior to the addition of fresh media (untreated), vehicle (0.15% DMSO), or 5 μM – 15 μM ebselen. Cells were counted using a hemacytometer and Trypan Blue exclusion to assess viability. Cells were counted on days 3 and 5, and “floaters” (disadhered and dead cells) were saved for determination of overall viability. Media was replaced on day 3 for the 5^th^ day time point; floaters were saved and added back to each well for counting on day 5. Viability was determined as [1-(# dead cells / (# live cells + # dead cells))*100]. Error is represented as the standard error of the mean. Significance was determined using paired *T*-testing for each time point compared to vehicle-treated cells.

### Trans-well invasion assays

2 × 10^4^ MIAPaCa-2, BXPC3, 786-O, or UOK117 cells were seeded in rehydrated 24-well invasion assay inserts containing 8 μm pores overlaid with Matrigel (Corning) in serum-free media; cells were adhered for 1 hour prior to addition of ebselen or vehicle. Inserts were incubated in wells containing complete media for 20 hours at 37°C. Non-invading cells were removed with cotton swabs and membranes were fixed with 100% methanol and mounted on slides with DAPI (Life Technologies). The total number of invading cells was determined by manual counting of DAPI-stained nuclei.

### Electrospray ionization mass spectrometry

2 μM rQSOX1 was incubated with 20 μM ebselen (10-fold excess) with or without 10 μM DTT substrate (added 5 minutes prior to mass analysis). rQSOX1 was analyzed intact by liquid chromatography-electrospray ionization-mass spectrometry (LC-ESI-MS) on a Dionex Ultimate 3000 HPLC equipped with a 1:100 flow splitter connected to a Bruker Maxis 4G quadrupole-time-of-flight (Q-TOF) mass spectrometer. A trap-and-elute form of LC-MS was carried out in which 15 μL samples were loaded at 10 μl/min in 80/20 water/acetonitrile containing 0.1% formic acid (loading solvent) onto a Bruker-Michrom protein captrap conFigured for bi-directional flow on a 6-port diverter valve. The trap was then rinsed with the HPLC loading pump at 10 μl/min for 40 min to completely remove PBS buffer salts. The flow over the captrap was then switched to the micropump, set at 2 μL/min, and ramped over 5 minutes from 80% water containing 0.1% formic acid (Solvent A)/20% acetonitrile (Solvent B) to 90% acetonitrile and held for 3 min.

The captrap eluent was directed to the mass spectrometer operating in positive ion, TOF-only mode, acquiring spectra in the *m/z* range of 300 to 3000 with a nominal resolving power of ~60, 000 m/ßm FWHM. ESI settings for the Agilent G1385A capillary microflow nebulizer ion source were as follows: End plate offset −500 V, capillary −3500 V, nebulizer nitrogen 2 bar, dry gas nitrogen 3.0 L/min at 225°C. Data were acquired in profile mode at a digitizer sampling rate of 4 GHz. Spectra rate control was by summation at 1 Hz.

rQSOX1 eluted over a period of about 1 minute; under the above conditions, rQSOX1 ranged in charge state from +32 to +60. Raw mass spectra were averaged across this timeframe, baseline subtracted and charge deconvoluted with Bruker DataAnalysis 4.1 charge deconvolution software to a mass range of 1000 Da on either side of any identified peak.

### Maleimide protection assay

2 μM rQSOX1 in 1X PBS (pH 7.5) was incubated with a 5-fold molar excess of ebselen or vehicle for 5 minutes at 25°C. AlexaFluor488-C5-maleimide (Life Technologies, Carlsbad CA) was added to a final concentration of 100 μM. Reactions were incubated for 30 minutes at 37°C, protected from light. Protein from each reaction was resolved on 12% polyacrylamide gels in non-reducing conditions. Gels were washed twice for 5 minutes in ddH_2_O and imaged under UV light. Gels were then stained with Coomassie R-250 for 15 minutes. Band intensities were analyzed by ImageJ, and are represented as “percent signal” of rQSOX1 pre-incubated with vehicle.

### Cyanylation and ammonia-based cleavage at free cysteines

Free cysteines on rQSOX1 were identified by treatment of recombinant enzyme with the sulfhydryl cyanylating reagent 1-Cyano-4-dimethylaminopyridinium tetrafluoroborate (CDAP) followed by ammonia-mediated N-terminal cleavage and analysis by MALDI-MS [39]. 100 pmol rQSOX1 was dissolved in 0.1 M citrate containing 6 M guanidine-HCl, pH 3.0. CDAP was added to a concentration of 25 mM from a freshly prepared 200 mM stock and incubated for 15 minutes at 25°C. Trifluoroacetic acid (TFA) was added to a concentration of 0.2%, and protein was purified using C18 ZipTips (Millipore); purified rQSOX1 was eluted with 90% ACN with 0.1% TFA in MilliQ H_2_O. Samples were dried and reconstituted in 6 M guanidine-HCl containing 1 M NH_4_OH, pH 11.5. Samples were incubated for 60 minutes at 37°C. Reactions were quenched by reducing the pH to 3.0 with citric acid. Disulfide bonds were reduced in 100 mM TCEP dissolved in ddH2O for 30 minutes at 37°C. 0.2% TFA was added, and C18 ZipTip purification was repeated as before. Samples were eluted with 3 ul 90% ACN/0.1% TFA directly onto MALDI targets; 2 μl saturated sinapinic acid in 33% ACN/0.4% TFA was added and samples air-dried.

### MALDI mass spectrometry

Masses of cyanylation/ammonia-induced protein cleavage products of rQSOX1 were determined by MALDI-MS on a Bruker Ultraflex-III MALDI mass spectrometer equipped with a Nd:YAG laser operating in positive-ion, delayed extraction linear mode, with ion source 1 at 25.00 kV, ion source 2 at 23.10 kV, lens at 7.50 kV, 150 ns delay, and 1 GS/s sample rate. Prior to acquisition of the mass spectra, the target mass range was externally calibrated using a mixture of calibrants obtained from Bruker Daltonics (Billerica, MA USA).

## SUPPLEMENTARY FIGURES


