# Supplementary material for: Ebselen inhibits QSOX1 enzymatic activity and suppresses invasion of pancreatic and renal cancer cell lines
Source: Oncotarget. 2015 Jun 1;6(21):18418–28. doi: 10.18632/oncotarget.4099 (PMC4621900; doi:10.18632/oncotarget.4099)
Supplement: Supplementary file 1 [file oncotarget-06-18418-s001.pdf]

SUPPLEMENTARY FIGURES

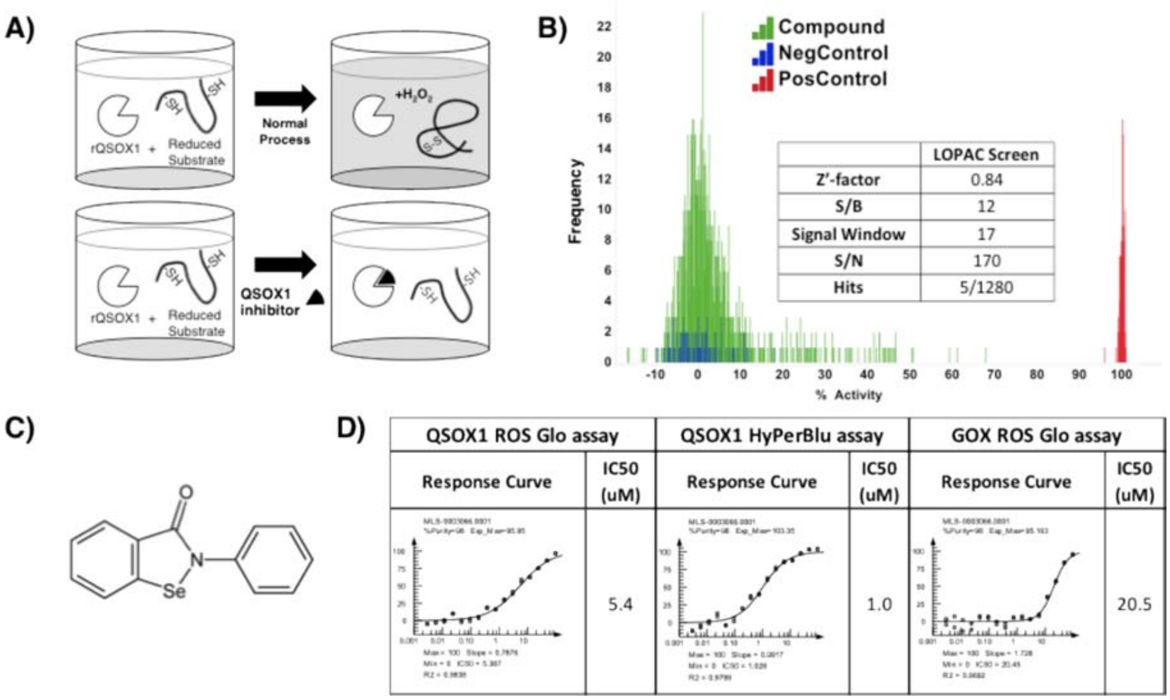

Supplementary Figure S1: High-throughput screen for QSOX1 inhibitors using LOPAC^{1280} identified ebselen as a QSOX1 inhibitor. **A.** Diagram of QSOX1 sulfhydryl oxidase activity reaction used in high-throughput screening and HVA activity assays. **B.** Distribution plot with primary HTS data showing positive (red bars) and negative (blue bars) controls, and compounds (green bars) at 12.5 µM concentration. Inset table summarizes plate statistics for HTS campaign. **C.** Structure of ebselen. **D.** Concentration-dependent inhibition curves for ebselen for QSOX1 (ROS Glo left, HyPerBlu middle) and GOx (ROS Glo right).

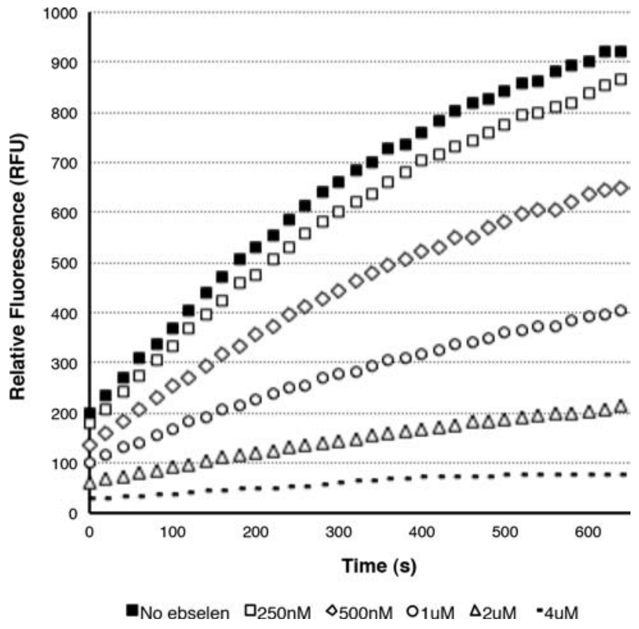

Supplementary Figure S2: Relative activity of 150 nM rQSOX1 with 300 µM DTT substrate in the presence of 250 nM – 4 µM ebselen. Ebselen was added to reactions at least 10 minutes prior to rQSOX1 addition. Reactions were monitored over a period of 10 minutes as described in methods.

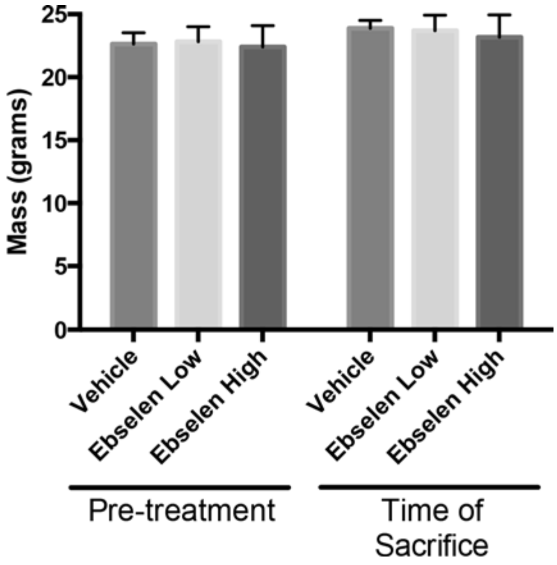

Supplementary Figure S3: Average weights of nude mice pre- and post-ebselen treatment. Nude mice were weighed prior to tumor implantation to the nearest 0.1 g. Mice were weighed again at the conclusion of the study, immediately after CO_2 asphyxiation. *N* = 5 per treatment group, error bars represent standard deviation.

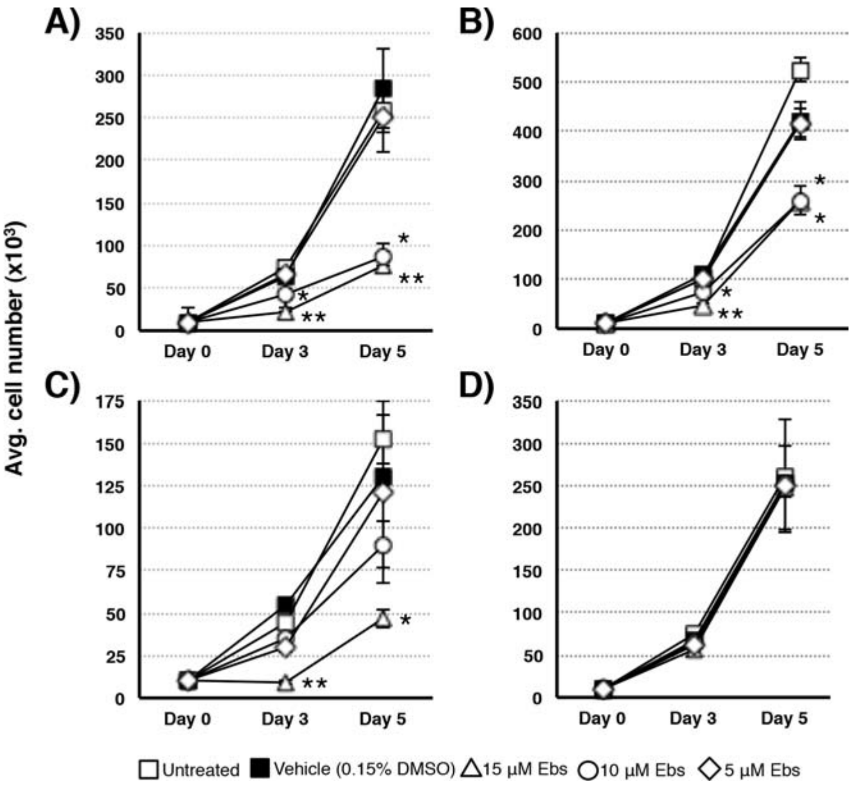

Supplementary Figure S4: Effect of ebselen on tumor cell line growth. Cell numbers for tumor cell lines grown in the presence or absence of ebselen or DMSO vehicle are shown. Viable cell numbers were determined manually using Trypan Blue. Each time point was performed in duplicate, and error is represented as SEM. Significance was determined for ebselen-treated cells compared to vehicle-treated cells using paired *T*-tests. Growth kinetics are shown for **A.** MIAPaCa-2, **B.** BXPC3, **C.** 786-O, and **D.** UOK117. *$p < 0.08$, **$p < 0.05$. At 5 days incubation, 10 µM ebselen decreased MIAPaCa-2 cell number by 70% compared to vehicle. For BXPC3, day 5 cell numbers were reduced by 38% for both 15 µM and 10 µM ebselen. 786-O and UOK117 were more resistant to ebselen. 15 µM ebselen decreased 786-O growth 79% and 65% at days 3 and 5, respectively. UOK117 growth was unaffected by ebselen treatment at all concentrations tested. It is important to note that the reduced cell numbers observed were not due to decreased viability from ebselen cytotoxicity (Figure S6).

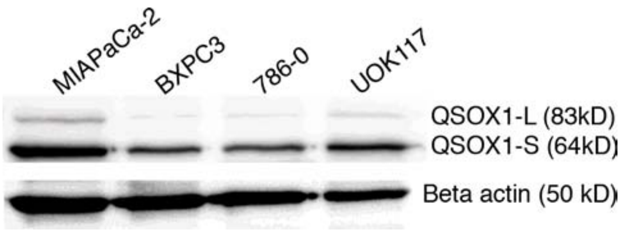

Supplementary Figure S5: Relative QSOX1 expression in pancreatic and renal cancer cell lines. 10 µg total protein loaded in 12% SDS-PAGE gels at 150 V. Proteins resolved and transferred onto PVDF membranes for one hour at 100 V. Membranes blocked for 1 hour with 1% BSA 0.1% TBST at room temperature on shaker. Membranes were incubated with 1:1000 anti-QSOX1 (ProteinTech), or 1:1000 anti-BACTN (Cell Signaling) followed by 1:10,000 goat anti-rabbit HRP for 1 hour at room temperature.

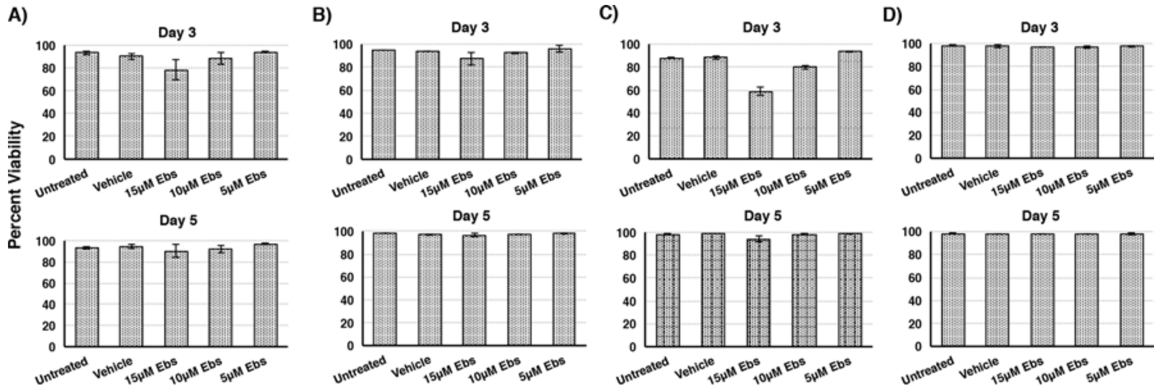

Supplementary Figure S6: Viability of tumor cell lines treated with ebselen. Total viability was determined at days 3 and 5 by Trypan Blue exclusion. Viability was calculated as [1-(# dead / (# dead + # alive))]*100. Error bars represent SEM. **A.** MIAPaCa-2, **B.** BXPC3, **C.** 786-O, **D**. UOK117. We observed no appreciable decrease in cell viability up to 15 µM at days 3 and 5, except for 786-O C. where viability at day 3 was 60%.

```
                  hsQSOX1 C165
H.sapiens         P--PACPPLEPAKLEEIDGFFARN-NEEYLALIFEKGGSYLGREVALDLSQHKGVAVRRV 218
P.troglodytes     P--PACPPLEPAKLEEIDGFFVRN-NKEYLALIFEKGGSYLGREVALDLSQHKGVAVRRV 219
G.gorilla         P--PACPPLEPAKLEEIDGFFARN-NEEYLALIFEKGGSYLGREVALDLSQHKGVAVRRV 218
P.albeii          P--PACPPLEPARLEEIDGFFARN-NEEYLALIFEKGGSYLGREVALDLSQHKGVAVRRV 221
M.mulatta         P--PACPPLEPAKLEEIDGFFARN-KEEYLALIFEKGGSYLGREVALDLSQHKGVVVRRV 218
M.musculus        P--PACPPLEPAKLNDIDGFFTRN-KADYLALVFEREDSYLGREVTLDLSQYHAVAVRRV 221
R.norvegicus      P--PACPPLEPAKLKDINEFFTRS-KAEYLALIFEREDSYLGREVTLDLSQFHAVAVRRV 221
B.taurus          P--PACPPLEPARLEEITGFFARN-NEEYLALIFEKEGSYLGREVTLDLSQHQGIAVRRV 219
G.gallus          P--PACPPLEPASAEEVRSFFHRN-TERYLALIFEQSNSFVGREVALDLLQYENVAVRRV 229
D.rerio           P--PACPPLETASEAEVHHFFPAN-NVKYLALVFENKKSYVGREVTLDLLQYENIAVRRV 227
A.carolinensis    P--PSCPPLEPISAAELHDFFQTN-SVTYLALIFEDENSFLGREVTLDMLQFENIAIRRV 210
X.laevis          P--PACPPLEPISAYEVEQFFITH-QEDYLALIFEEASMYIGRETALDMVQYEGVSVRRV 210
C.capitata        PNYPNLNSLTENEAKTLNKVFTTDSNKKFVILVYEPENSTVGLESILHFHLWSDVQVRRI 235

                            hsQSOX1 C237
H.sapiens         LNTEANVVRKFGVTDFPSCYLLFR-NGSVSRVPVLMESRSFYTAYLQRLSGLTRE----- 272
P.troglodytes     LNTEANVVRKFGVTDFPSCYLLFR-NGSVSRVPVLMESRSFYTAYLQRLSGLTRE----- 273
G.gorilla         LNTEANVVRKFGVTDFPSCYLLFR-NGSVSRVPVLMESRSFYTAYLQRLSGLTRD----- 272
P.albeii          LNTEANVVRKFGVTDFPSCYLLFR-NGSVSRVPVLMESRSFYTAYLQRLSGLTRE----- 275
M.mulatta         LNTEADVVRKFGVTDFPSCYLLFR-NGSVSRVPVLMESRSFYTAYLQRLSGLTRE----- 272
M.musculus        LNTESDLVNKFGVTDFPSCYLLLR-NGSVSRVPVLVESRSFYTSYLRGLPGLTRD----- 275
R.norvegicus      LNSESDVVSKFAVTDFPSCYLLLR-NGSVSRVPVLVESRPFYTSYLRGLPGLTRE----- 275
B.taurus          LNTERDVVNRFGVTNFPSCYLLSR-NGSFSRVPALTESRSFYTTYLRKFSGSTRG----- 273
G.gallus          LSSEEELVEKFGVTTFPSAYLLLR-NGSFSRLPVHAEARSFYTYYLQTLSGVTRG----- 283
D.rerio           LDTETNLVSRFGVTEFPSCYLYDS-SGNITRLKVLKEARTFYSYALQRLPGVVRTG---- 282
A.carolinensis    LQTNEELVKRFNVTSFPSGFLLVN-NGSCSSIPVNADFRPFYRSFLQSLPNVFRGN---- 265
X.laevis          HRDQDDIVNKFQIPSFPALVLLCK-NGSNTIVNMVEDTRSSYTNFLRSLPGVRKG----- 264
C.capitata        N--DINLANTFQIDGGKHKIATVDPQGNVVPYGVTEDTPQAYTATIENLLTAQGFTPRTV 293
```

Supplementary Figure S7: Multi-species alignment of region in the vicinity of C165 and C237 in human QSOX1. Protein sequences for QSOX1 were obtained from UniProt from Homo sapiens (O00391), Pan troglodytes (H2Q0P8), Gorilla gorilla (G3R3B5), Pongo albeii (H2N4I1), Macaca mulatta (F7HHU1), Mus musculus (Q8BND5), Rattus norvegicus (Q6IUU3), Bos Taurus (F1MM32), Gallus gallus (F1NYK2), Danio rerio (B0UXN0), Anolis carolinensis (G1K901), Xenopus laevis (A0JPG9), Ceratitis capitata (W8C0Z9); UniProt ID numbers in parentheses. Seqeuences were aligned using ClustalW2 (40). Conserved cysteines at human positions C165 and C237 are bolded and colored blue. The cysteine at human position C165 is conserved in sequence from all vertebrate species analyzed, but not in the fruit fly Ceratitis capitata. Human C237 is less conserved, not present in vertabrates G. gallus, A. carolinensis, X. laevis, and the invertebrate C. capitata.

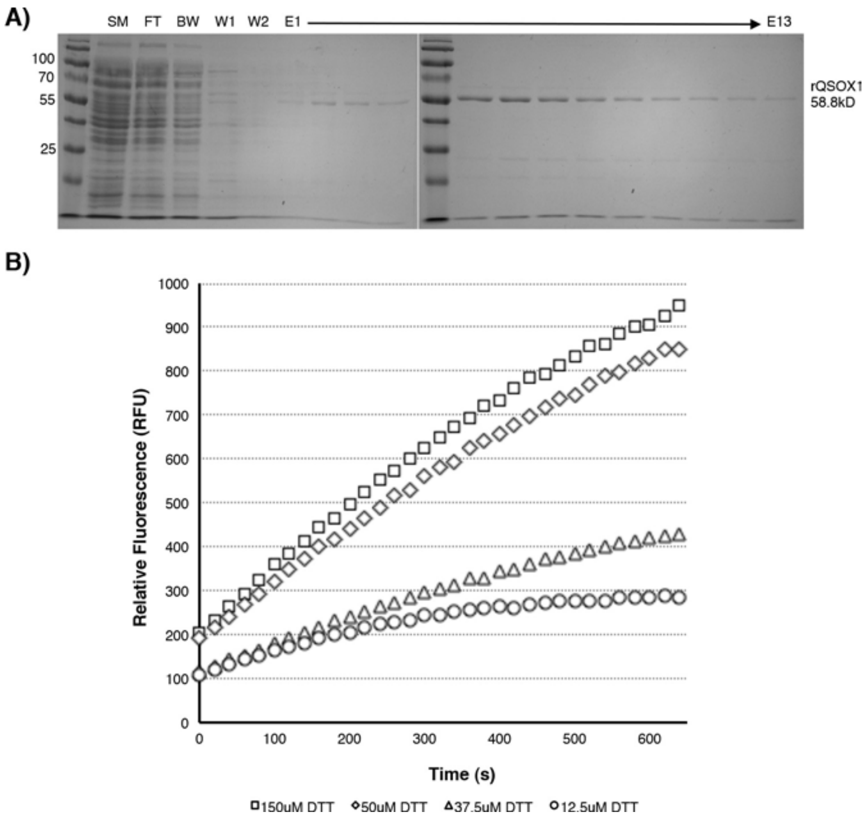

Supplementary Figure S8: Purification of active rQSOX1. A. Elution profile of rQSOX1. SM = starting material (lysate), FT = NI − NTA column flowthrough, BW = binding buffer wash, W1 = wash #1, W2 = wash #2, E1-E13 = elution fractions. **B.** Activity of rQSOX1 reacted with varying DTT substrate concentrations. As described in methods section, 150 uM, 50 uM, 37.5 uM, and 12.5 μM DTT was used in HVA-based activity assays with 150 nM rQSOX1. Total relative fluorescence was monitored over a 10 minute period.

Sequence coverage of trypsin-digested rQSOX1 (MALDI):

MGHHHHHHHMSALYSPSDPLTLLQADTVRGAVLGSRSAWAVEFFASWCGHCIAFAPTWKALAEDV
KAWRPALYLAALDCAEETNSAVCR**DFNIPGFPTVR**FFKAFTKNGSGAVFPVAGADVQTLRERLID
ALESHHDTWPPA**(C)**PPLEPAK**LEEIDGFFARNNEEYLALIFEK**GGSYLAREVALDLSQHKGVAV
RR**VLNTEANVVRK**FGVTDFPS**(C)**YLLFRNGSVSR**VPVLMESRSFYTAYLQR**LSGLTREAAQTTV
APTTANK**IAPTVWK**LADR**SKIYMADLESALHYILRIEVGRFPVLEGQR**LVALKKFVAVLAK**YFPG**
RPLVQNFLHSVNEWLKRQKRNKIPYSFFKTALDDRKEGAVLAK**KVNWIGCQGSEPHFR**GFPCSLW
VLFHFLTVQAARQNVDHSQEAAK**AKEVLPAIR**GYVHYFFGCRDCASHFEQMAAASMHR**VGSPNAA**
VLWLWSSHNRVNARLAGAPSEDPQFPKVQWPPRELCSACHNER**LDVPVWDVEATLNFLKAHFSPS**
NIILDFPA

Sequence coverage of trypsin-digested rQSOX1 (Orbitrap, LC-MS/MS):

MGHHHHHHHMSALYSPSDPLTLLQADTVRGAVLGSRSAWAVEFFASWCGHCIAFAPTWK**ALAEDV**
KAWRPALYLAALDCAEETNSAVCR**DFNIPGFPTVRFFKAFTKNGSGAVFPVAGADVQTLR**ERLID
ALESHHDTWPPA(C)PPLEPAK**LEEIDGFFARNNEEYLALIFEK**GGSYLAR**EVALDLSQHKGVAV**
RRVLNTEANVVRKFGVTDFPS(C)YLLFRNGSVSR**VPVLMESRSFYTAYLQRLSGLTREAAQTTV**
APTTANKIAPTVWKLADRSK**IYMADLESALHYILRIEVGRFPVLEGQRLVALKKFVAVLAK**YFPG
RPLVQNFLHSVNEWLKRQKR**NKIPYSFFKTALDDRKEGAVLAKK**VNWIGCQGSEPHFRGFPCSLW
VLFHFLTVQAAR**QNVDHSQEAAKAKEVLPAIR**GYVHYFFGCRDCASHFEQMAAASMHR**VGSPNAA**
VLWLWSSHNRVNARLAGAPSEDPQFPKVQWPPRELCSACHNER**LDVPVWDVEATLNFLK**AHFSPS
NIILDFPA

Supplementary Figure S9: Mass spectral analysis of trypsin-digested rQSOX1. Peptides identified from **A.** MALDI, or **B.** LC-MS/MS analysis are bolded and underlined. Underlined (but not bolded) residues represent redox-active C-X-X-C motifs. Cysteines in parentheses, C165 and C237, were identified as ebselen-binding cysteines.
